# Novel Positive Allosteric Modulators of µ Opioid Receptor—Insight from In Silico and In Vivo Studies

**DOI:** 10.3390/ijms21228463

**Published:** 2020-11-11

**Authors:** Damian Bartuzi, Ewa Kędzierska, Agnieszka A. Kaczor, Helmut Schmidhammer, Dariusz Matosiuk

**Affiliations:** 1 Department of Synthesis and Chemical Technology of Pharmaceutical Substances with Computer Modeling Laboratory, Faculty of Pharmacy, 4A Chodźki St, PL-20093 Lublin, Poland; dariusz.matosiuk@umlub.pl; 2 Department of Pharmacology and Pharmacodynamics, Faculty of Pharmacy, 4A Chodźki St, PL-20093 Lublin, Poland; ewa.kedzierska@umlub.pl; 3 School of Pharmacy, University of Eastern Finland, Yliopistonranta 1, P.O. Box 1627, FI-70211 Kuopio, Finland; 4 Department of Pharmaceutical Chemistry, Institute of Pharmacy and Center for Molecular Biosciences Innsbruck (CMBI), University of Innsbruck, Innrain 80-82, AT-6020 Innsbruck, Austria; helmut.schmidhammer@uibk.ac.at

**Keywords:** allosteric modulation, antinociceptive compounds, behavioral studies, molecular dynamics, opioid receptors

## Abstract

Opioids are the drugs of choice in severe pain management. Unfortunately, their use involves serious, potentially lethal side effects. Therefore, efforts in opioid drug design turn toward safer and more effective mechanisms, including allosteric modulation. In this study, molecular dynamics simulations in silico and ‘writhing’ tests in vivo were used to characterize potential allosteric mechanism of two previously reported compounds. The results suggest that investigated compounds bind to μ opioid receptor in an allosteric site, augmenting action of morphine at subeffective doses, and exerting antinociceptive effect alone at higher doses. Detailed analysis of in silico calculations suggests that first of the compounds behaves more like allosteric agonist, while the second compound acts mainly as a positive allosteric modulator.

## 1. Introduction

Effective pain management is one of the main challenges of the current medicine. The most powerful antinociceptive agents are opioids which act through µ opioid receptor (MOR). Although MOR activation relieves acute and severe pain, it is connected with several side effects [1]. Firstly, the rewarding properties of opioids can lead to addiction and pose a risk of overdosing [2]. Secondly, these drugs have several other side effects, such as sedation, dizziness, nausea, vomiting, constipation, and respiratory depression [3]. To overcome these limitations of opioid drugs, efforts are made to develop safer medications. One approach is to search for G protein biased MOR agonist, such as TRV-130 (oliceridine) which has been recently approved by FDA for the management of acute pain severe enough to require an intravenous opioid analgesic [4]. Another possibility is to focus on MOR positive allosteric modulators (PAMs) instead of orthosteric ligands.

Allosteric ligands (with the exception of silent or neutral allosteric ligands) possess the ability to modulate G protein-coupled receptors (GPCRs) function by binding to receptor regions away from the orthosteric binding site. Allosteric modulators usually bind to receptor areas with a low degree of conservation between GPCR subtypes [5]. This binding specificity could be the basis for the design of more selective drugs. Another mechanism that may lead to selectivity is via selective cooperativity with the orthosteric ligand, which is possible even when the allosteric site is similar for receptor subtypes [6,7]. Furthermore, effects of allosteric modulators are saturable [8] and thus such an approach may make it possible to solve the problem of drug dependence, overdose risk and other adverse effects linked with classical orthosteric drugs. Additionally, the fact that allosteric modulators can function together with ligands interacting at the orthosteric binding site makes drugs exploiting this phenomenon especially useful when treatment can be achieved by enhancing or decreasing an endogenous signal. Next, due to the lack of chronic activation of a receptor, tachyphylaxis may be limited, which enables to overcome the problem of diminishing therapeutic efficacy resulting from chronic administration of many orthosteric agonists [7,8]. Finally, allosteric modulators can bias receptor signaling to a specific signaling readout by restricting receptor conformations to engage effector/accessory proteins [8]. By doing it, functionally biased allosteric modulators can be applied in restoring balanced signaling in systems where disease has changed downstream signaling, or even establish “new” functional receptor systems with unique signaling capability [8]. Novel allosteric modulators of GPCRs are thus providing fundamental advances in the development of GPCR ligands with high subtype selectivity [9] and novel modes of efficacy that have not been possible with traditional approaches [10]. This understanding creates exciting opportunities, as well as unique challenges, in the optimization of novel therapeutic agents for disorders of the central nervous system [10].

**Figure 1:**
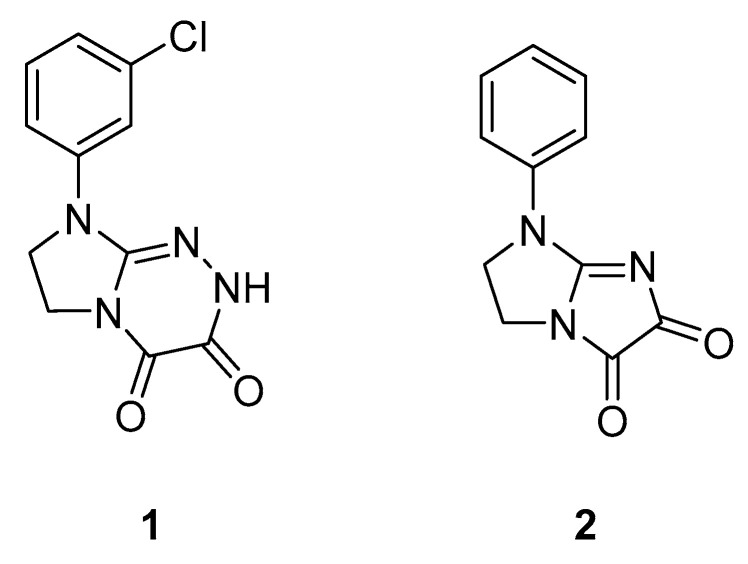
Structural formulas of the investigated compounds.

Only few MOR PAMs are known (for review see [11]). These incudes BMS986121, BMS986122 [12], as well as MS1 [13] and several of its analogs were identified mainly through large library screening. We have made an effort to develop novel antinociceptive compounds with confirmed [14,15,16] or probable [17,18,19,20,21] activity through MOR. All these compounds fulfill non-classical MOR opioid receptor pharmacophore model [15], further extended by our group [21] which involves a base, a hydrophobic and aromatic moiety or hydrogen bond acceptor, hydrophobic and aromatic groups. Two adjacent hydrogen bonds acceptors fit in the pharmacophore suggested by Bisignano et al. [13]. Selected compounds from all the series exhibit antinociceptive properties in vivo reversed by naloxone which suggests that their antinociceptive activity is mediated by MOR. In addition, for selected compounds from the compound series described in references [14,15,16] EC_50_ values to MOR were determined. In particular, we found that two previously reported compounds, **1** [21] and **2** [16] (Figure 1) probably are MOR PAMs. These compounds have antinociceptive properties in the ‘writhing’ test in mice [16,21] and compound **2**, partially displacing naloxone from MOR, is characterized by EC_50_ value to this receptor of 14 µM [16]. Moreover, it is characterized by EC_50_ value to serotonin 5-HT_2A_ receptor of 8.4 µM and benzodiazepine (BZD) of 31.0 µM receptor. Here we used further in vivo studies and extensive molecular modeling studies to verify the hypothesis about possible allosteric properties of both compounds at MOR.

## 2. Results

### 2.1. In Vivo Studies

#### 2.1.1. Effect of Pre-Treatment with Cyprodime (CYP) on the Antinociceptive Effect of New Compounds 1 (0.0125 LD_50_) and 2 (0.05 LD_50_) in the ‘Writhing’ Test in Mice

**Figure 2:**
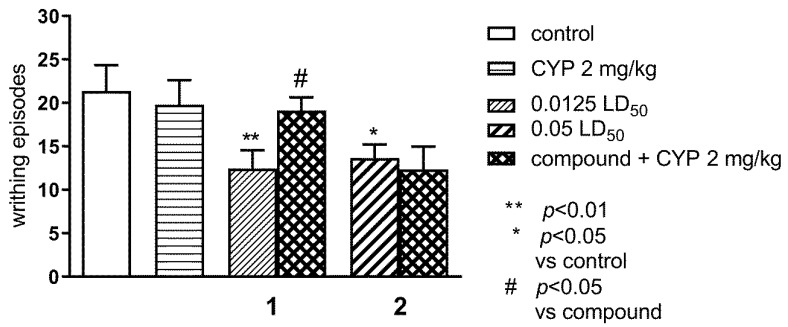
Effect of pre-treatment with CYP on the antinociceptive effect of new compounds **1** (0.0125 LD50) and **2** (0.05 LD50) in the ‘writhing’ test in mice. The tested compounds **1** and **2** were injected i.p. 60 min before the test and CYP, 2 mg/kg, s.c. 25 min before the test. Data are expressed as mean ± SEM values. Groups of 10–12 animals were tested. ** *p* < 0.01; * *p* < 0.05 vs. control vehicle-treated group, # *p* < 0.05 vs. respective compound (Bonferroni’s test).

The effect of CYP on the antinociceptive effects of compounds **1** and **2** assessed in the ‘writhing’ test in mice is shown in Figure 2. One-way ANOVA showed significant changes in the number of writhing episodes of mice after the administration of compounds **1** and **2** and co-administration of CYP (2 mg/kg) with the tested compounds (F(5,64) = 3.122; *p* < 0.05). Bonferroni’s post hoc test revealed that CYP reversed antinociceptive effect of compound **1** (*p* < 0.05) at the dose of 0.0125 LD_50_.

#### 2.1.2. Effect of Combined Administration of Morphine (0.5 mg/kg i.p.) and Tested Compounds 1 0.00625 LD_50_) and 2 (0.0125 LD_5_0) in the ‘Writhing’ Test on Mice

**Figure 3:**
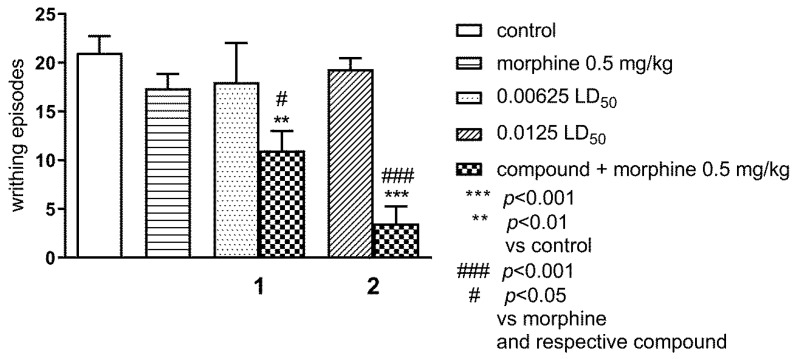
Effect of combined administration of morphine (0.5 mg/kg i.p.) and tested compounds **1** 0.00625 LD_50_) and **2** (0.0125 LD_50_) in the ‘writhing’ test in mice. The tested compounds **1** and **2** were injected i.p. 60 min before the test and morphine 0.5 mg/kg, s.c. 25 min before the test. Data are expressed as mean ± SEM values. Groups of 10-12 animals were tested. *** *p* < 0.001, ** *p* < 0.01; vs. control vehicle-treated group, ### *p* < 0.001, # *p* < 0.05 vs. morphine and respective compound (Bonferroni’s test).

The results in Figure 3 demonstrate that both morphine (0.5 mg/kg) and tested compounds **1** and **2**, administered alone at ineffective doses **1**: 0.00625 LD_50_ and **2** 0.0125 LD_50_, respectively, had no effect on the writhing episodes. However, simultaneous administration of morphine and tested compounds **1** and **2** at the above-mentioned doses, resulted in a statistically significant reduction of the writing episodes as compared to the morphine group and respective compound (*p* < 0.05 and *p* < 0.001), as well as to the control group (*p* < 0.01 and *p* < 0.001); ANOVA: F(5,44) = 9.070; *p* < 0.0001; Bonferroni’s test.

### 2.2. In Silico Studies

In our previous studies, we addressed mechanisms of allosteric modulation of MOR by negative and positive modulators known from literature [22,23,24]. Results of these simulations were further validated by in silico and in vitro studies of independent research groups [25,26,27]. Therefore, we decided to use this validated framework for investigation of putative allosteric action of compounds **1** and **2**, taking advantage of considerable in-house frame of reference available.

**Figure 4:**
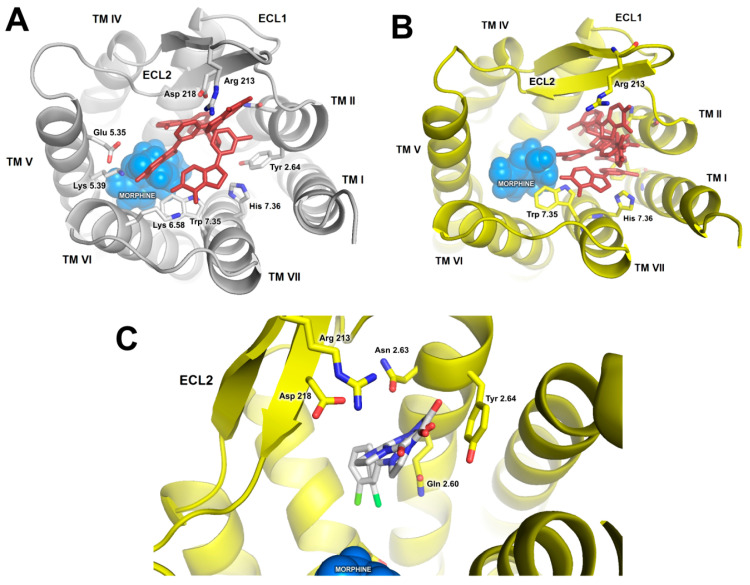
Possible binding modes of compound **1** in MOR. (**A**) Representative poses of the most abundant docking clusters used for short, 50 ns MD simulations. (**B**) Superposition of final ligand poses after short MD simulations. (**C**) In several simulations, compound **1** consistently drifted to similar poses, with phenyl moiety immersed in the aromatic pocket below the extracellular loop 2 (ECL2), and a dione part interacting with polar residues from the loop.

The first step was flexible docking of investigated compounds to the extracellular part of the receptor. The docking was performed in presence and in absence of an orthosteric ligand. Morphine was chosen for this purpose, since it was used in the in vivo part of the study. Subsequently, best-scoring representatives of docking clusters of compound **1** (Figure 4A) were used for short, duplicated 50 ns molecular dynamics (MD) simulations, in order to assess stability of the poses. In most cases, ligand molecules drifted to the same region of the receptor (Figure 4B). In particular, final poses of all simulations were located in direct proximity of TM2, and most of them (4 of 6 short simulations) showed the investigated compounds being anchored by the phenyl moiety interacting with the hydrophobic pocket composed of Trp 135, Val 3.28, Ile 3.29 and cysteine bridge between Cys 3.25 ad Cys 219, below the extracellular loop 2 (ECL2), and ketone groups interacting with arginine or aspartate from ECL2, either directly or through water molecules (Figure 4C). Since these poses were also energetically favorable, they were chosen for longer simulations. Docking results for compound **2** were less diverse and mostly consisted of poses located below ECL2, in agreement with MD results for compound **1**. Therefore, the best scored pose was used in further steps.

In the next step, 400 ns simulations were performed in various ligand configurations. To provide appropriate reference, we performed simulations of MOR in complex with an orthosteric partial agonist (morphine) and an orthosteric antagonist (naloxone). Additionally, trajectories of MOR in complex with a full agonist ((R)-methadone) from the previous study [24] were added to analysis for comparison. Complexes of MOR with compounds **1** and **2** were simulated in presence of morphine in the orthosteric site. Additionally, due to interesting preliminary in silico results and relatively low doses needed for nociceptive activity in vivo, additional simulations of compound **1** in complex with MOR without any orthosteric compounds were performed. Visual analysis performed on trajectories revealed both similarities and differences in behavior of compound **1** and compound **2** in the allosteric binding pocket. Both compounds established frequent direct contacts with Tyr 2.64 through the heterocyclic rings, and preferred location of the phenyl moiety was the pocket between Trp 135, Val 3.28, Ile 3.29. However, chlorine substituent seems to prevent the phenyl moiety from stable binding in the hydrophobic aromatic pocket under ECL2. The compound **1** is therefore more shallowly anchored. Visual analysis of trajectories shows increased motility of the compound **1** in the allosteric pocket, especially regarding the heterocyclic part. To confirm this observation, we measured the ligand root mean square fluctuation (RMSF), with 7TM bundle Cα atoms as fitting reference. RMSF values, presented in Table S1, confirm higher motility of compound **1** in presence of morphine in the orthosteric pocket, when compared to values obtained for compound **2**. To further investigate this phenomenon, we measured root mean square deviation (RMSD) values for both investigated compounds, calculated between a given pose and a pose 500 ps before in trajectory. The evolution of RMSD in time reveals several peaks corresponding to changes in compound 1 conformation, as shown in Table S2.

Principal component analysis (PCA) was used to sift relevant relationships. Trajectories of 7-transmembrane (7TM) domain obtained from simulations were extracted and concatenated, so that PCA of all simulations was done in the same conformational space. Several PCA rounds were performed, for the whole 7TM domain and for particular helices separately. Heavy atoms of all residues of 7TM bundle except the hydrophobic residues located at the protein-membrane interface were considered in the analysis, including sidechains. Sidechains of residues located at the protein-membrane interface were ignored to remove noise. PCA was performed on whole trajectories. To clarify presentation of the results, conformational space explored through 50 last ns only is presented in figures.

**Figure 5:**
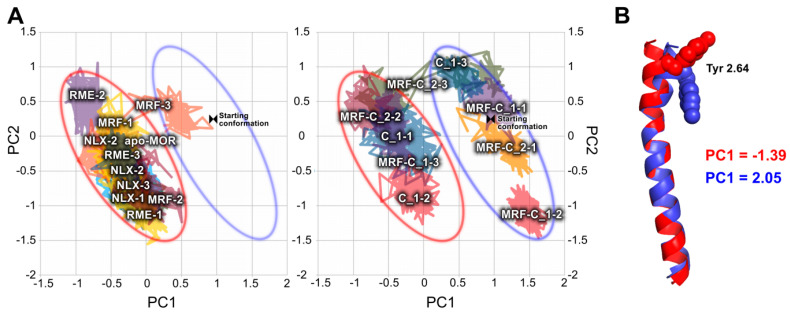
PCA of TM2. (**A**) Conformational space occupied by investigated complexes through last 50 ns of simulations in a common space. For clarity, data is split into two panels. Left panel: complexes with orthosteric ligand only; right panel: complexes containing compound **1** or compound **2**. Ellipses were added to the graphs to facilitate comparison. Color-coding of ellipses corresponds to the part B of the figure. (**B**) Projections of extreme PC1 values on trajectories, depicting main conformational differences between groups presented in the part A of the figure. Color-coding of the legend corresponds to colors of the helices.

PCA calculations made for the transmembrane helix 2 (TM2) and 7 (TM7) yielded interpretable results. As shown in Figure 5, presence of compound **1** or compound **2** affects conformation of TM2. In all complexes without investigated compounds bound, area of the low first principal component (PC1) values was occupied. On the other hand, introduction of these compounds in several cases resulted in exploration of completely new conformational space. Comparison of projections of extreme values of PC1 on trajectories revealed that the main difference is conformation of Tyr 2.64 (Ballesteros-Weinstein notation [28]). While in absence of any putative modulators this residue tends to protrude outwards of the receptor, in their presence it frequently points toward protein interior.

**Figure 6:**
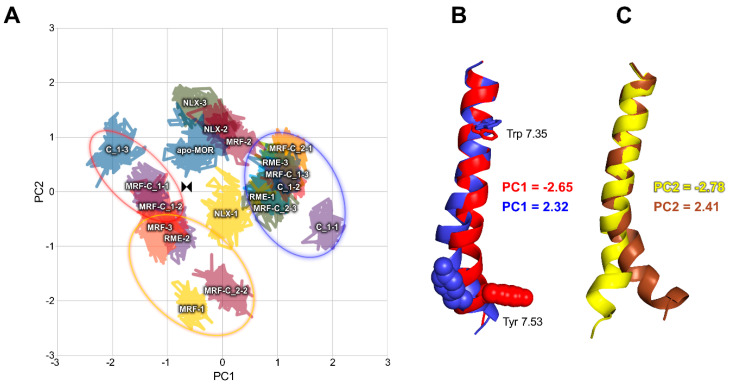
PCA of TM7. (**A**) Conformational space occupied by investigated complexes through last 50 ns of simulations in a common space. Ellipses were added to the graphs to facilitate referring to projections on trajectories. Color-coding of ellipses corresponds to the part B and C of the figure. (**B**) Projections of extreme PC1 values on trajectories, depicting main conformational differences between groups presented in the part A of the figure. Color-coding of the legend corresponds to colors of the helices. (**C**) Projections of extreme PC2 values on trajectories, depicting main conformational differences between groups presented in the part A of the figure. Color-coding of the legend corresponds to colors of the helices.

Results of PCA calculations for TM7 are presented in Figure 6. In this case, relationships could be observed across both PC1 and PC2. Complexes containing investigated compounds tend to assume conformations described by extreme values of PC1, and PC2 values around zero. The same tendency is observed for two of three systems containing full agonist, (R)-methadone. Meanwhile, complexes of MOR with morphine or naloxone assumed PC1 values near zero, and a broad range of PC2 values, with naloxone prevailing in above-zero region, and morphine in below-zero area. Projections of extreme values of these principal components on trajectories revealed that these PCs mostly describe TM7 bending and rotation, as well as Trp 7.35 and Tyr 7.53 conformation, which is in line with our previous simulations of MOR allosteric modulators [23,24]. Interestingly, most simulations of compound **1** bound alone (without orthosteric ligands) tend to occupy the same conformational space as simulations of full agonist, which remains consistent with relatively low doses required for antinociceptive effect in vivo.

**Figure 7:**
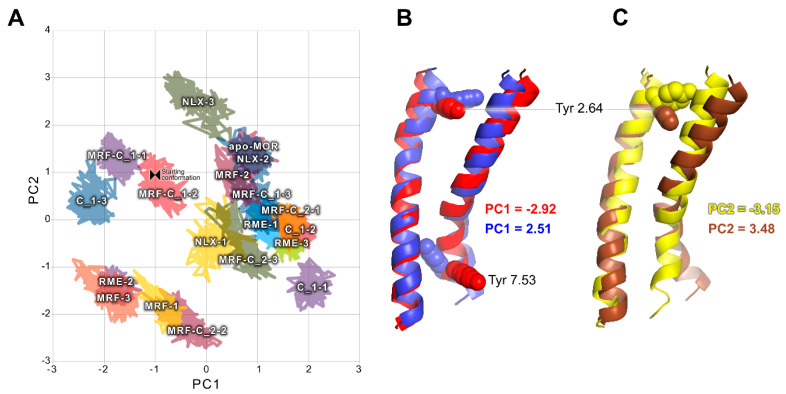
Joint PCA of TM2 and TM7. (**A**) Conformational space occupied by investigated complexes through last 50 ns of simulations in a common space exhibits large similarity to PCA calculated for TM7 alone. (**B**) Projections of extreme PC1 values on trajectories. Color-coding of the legend corresponds to colors of the helices. (**C**) Projections of extreme PC2 values on trajectories. Color-coding of the legend corresponds to colors of the helices.

To obtain a more general view on statistics of ligand-dependent conformational changes, PCA for TM2 and TM7 together was calculated (Figure 7). Topology of conformational space described by first two PCs exhibits apparent similarity to PCA calculated for TM7 alone. Projections of extreme values of these PCs on trajectories show that the most pronounced conformational changes involve Tyr 2.64 and Tyr 7.53 residues.

**Figure 8:**
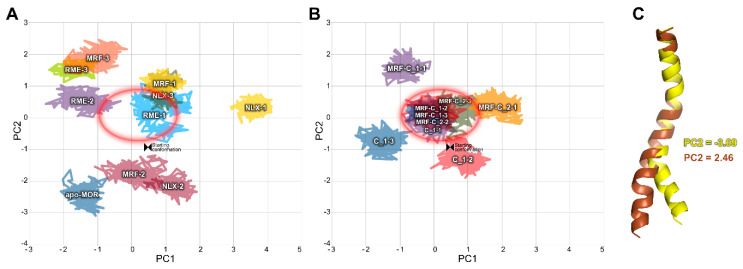
PCA of TM6. (**A**,**B**) Conformational space occupied by investigated complexes through last 50 ns of simulations in a common space. The data were split in two panels for clarity. The red ellipse is added to facilitate comparisons. (**C**) Projections of extreme PC2 values on the trajectories. Color-coding of the legend corresponds to colors of the frames.

Another relationship was observed in PCA calculated for TM6 alone (Figure 8). Most complexes containing compound **1** or compound **2** assumed a well-defined conformation in terms of PC1 and PC2. Interestingly, the values of PC2 characteristic for these complexes correspond to values obtained for two (R)-methadone-containing complexes.

**Figure 9:**
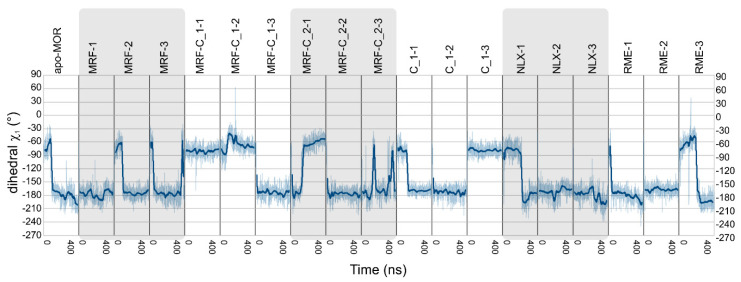
Values of χ_1_ dihedral of Tyr 2.64 residue measured in all simulations. Transparent line represents actual values, opaque thick line was added to facilitate analysis and represents a moving average.

**Figure 10:**
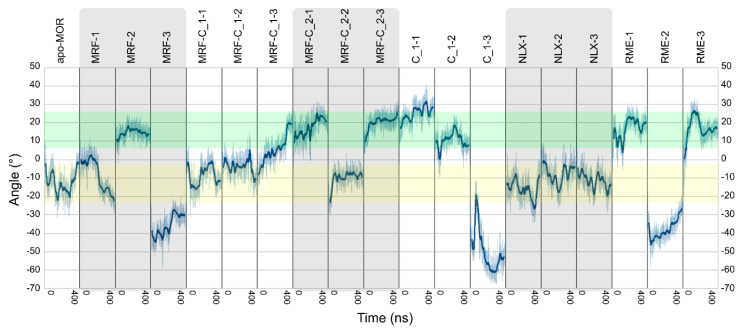
Rotation of intracellular part of TM7, measured below Asn 7.49 residue. Full agonist-like values are highlighted in green, antagonist-like values highlighted in yellow. Transparent line represents actual values, opaque thick line was added to facilitate analysis and represents a moving average.

PCA was calculated on covariance matrix of Cartesian coordinates. To check how the statistical analysis corresponds to actual conformations in the trajectories, angles and dihedrals pointed by PCA were measured in unmodified trajectories. The most apparent relationship between binding of compounds **1** or **2** and the receptor conformation was observed in case of χ_1_ dihedral of Tyr 2.64 residue (Figure 9, Table S3) and rotation of intracellular part of TM7 (Figure 10, Table S4). In the former, in the absence of any modulator, χ_1_ of the residue oscillates around −180° for most of the simulation time, while in presence of compound **1** it is shifted to around −60° in two of three simulations. Moreover, compound **1** was able to induce such change and keep it stable even in absence of morphine in one of three simulations. This effect occurs probably due to direct interactions of the compounds with Tyr 2.64 residue, as they remain in spatial proximity as depicted in Figure 4. In case of TM7 rotation, interpretation of the results is less straightforward, but careful analysis can lead to some conclusions. Apparently, compound **2** altered behavior of morphine and made it very similar to (R)-methadone, especially considering particular random seed series. Compound **1** alone also shifted TM7 rotation to values more characteristic to the full agonist. In turn, compound **1** bound together with morphine made TM7 assume ‘intermediate’ conformation, with angle values between those seen in full agonist- and antagonist-bound receptor. Hypothetically, as TM2 and TM7 are adjacent, ligand interactions with Tyr 2.64 could be the trigger of the allosteric signal migrating downwards to the intracellular part of TM7, affecting activation.

In PCA, residue Tyr 7.53 also seemed to be affected, similarly as in our previous studies on MOR modulation, so its dihedrals were also measured (Figure S1 in Supplementary Materials). Its conformation has shown that compound **1** is able to induce agonist-bound-like changes in the receptor, even in absence of orthosteric ligands, while in presence of morphine, compound **2** provides more stable arrangement of the residue.

## 3. Discussion

The tested compounds exhibited weak or moderate acute toxicity (as LD_50_): over 2000 mg kg^−1^ for compound **1** and 180 mg kg^−1^ for compound **2** and antinociceptive activity in the writhing test [16,21]. This test remains very commonly used, since it is a chemical method useful for sifting molecules whose pharmacodynamics properties are unknown, based on chemical stimulation by i.p. administration of agents that irritate serous membranes and provokes characteristic abdominal contractions. The writhing test lacks specificity but it is very sensitive, predictive and the closest in nature to clinical pain. It is a model of visceral or peritoneal pain, although it does not preclude central mechanisms of antinociception [29]. Strong impact on behavior of mice in this test was observed for compounds **1** and **2** at a wide range of doses—up to 0.00625 LD_50_ and 0.0125 LD_50_, respectively [16,21]. In this study, analgesic activity of tested compounds without or in the presence of morphine and CYP, was quantified in the writhing tests. An important finding of the current study is the observation that the antinociceptive effect of compound **1** was reversed by CYP, an µ-opioid antagonist, which indicates possible interaction with µ-opioid receptors. In addition to previous studies, where binding of compound **2** to μ opioid receptor was confirmed with radioligand binding assay, showing EC_50_ of 14 μM, [16] and considering structural similarity, this suggests that both compounds act at μ opioid receptor. Enhancement of the effect of morphine for both studied compounds seems to additionally confirm the participation of opioid receptors (mainly μ) in their antinociceptive effect. It has to be stressed, however, that radioligand binding of compound **2** at BZD receptor revealed moderate activity, with EC_50_ of 31 μM, [16] which could also be partially responsible for the reduction of writhing episodes [30]. In our studies, the tested compounds injected at ineffective doses were able to potentiate the antinociceptive action of morphine (at a subeffective dose of 0.5 mg/kg) in the writhing test by decreasing significantly the number of writhing episodes.

In the in silico part of the study, particular emphasis was put on native-like conditions in simulation boxes. The receptor was simulated in complex with G protein, and composition of the lipid bilayer reflected properties of lipid rafts [31]. This methodology proved to be successful in previous studies on other MOR modulators [22,23,24] and other subtle allosteric effects in other GPCRs [32]. Notably, investigation of compounds **1** and **2** yielded results compatible with in vitro and in vivo observations, as well as with studies of other modulators of opioid receptors. In particular, the allosteric binding pocket suggested by docking and MD simulations is largely analogous to that suggested for BMS986122 [23] and BMS986187 [27], and is located within a region of proposed common allosteric pocket of opioid receptors [26]. Moreover, area of a cavity below the extracellular loop 1 (ECL1) and ECL2 seems to be important in binding of allosteric or bitopic ligands also in related receptors, including dopamine receptors [33,34], which also belong to rhodopsin-like GPCRs and, similarly to opioid receptors, possess a conserved Asp 3.32 residue within the orthosteric binding pocket, responsible for interactions with basic moieties of ligands. Notably, while our study suggests a role of Tyr 2.64 in mechanisms of allosteric modulation by the reported compounds, Glu 2.65 residue was proven to play a key role in binding and action of SB269652 modulator in D_2_ and D_3_ dopamine receptors [35]. On one hand, this serves as additional validation of the location of the allosteric pocket identified in this study. On the other hand, it supports the concept of common conserved allosteric pathways in GPCRs [9,36].

Analysis of in silico results allows drawing general conclusions about mechanisms involved in action of investigated compounds. The low dose required for antinociceptive action of compound **1** at the level of 0.00625 LD_50_ (Figure 2) is reflected in its ability to induce and retain active-like receptor conformations, even in absence of any orthosteric ligand (Figure 9 and Figure 10, Figure S1 in Supplementary Information). However, in the writhing test, it seems to have weaker PAM properties (Figure 3), which is reflected in intermediate conformations of TM7 induced in MD simulations (Figure 10) and relative instability of Tyr 7.53 conformation induced by this compound in presence of morphine (Figure S1). In turn, stronger PAM properties of compound **2** (Figure 3) are reflected in inducing full agonist-like arrangement of TM7 (Figure 10) and stable Tyr 7.53 conformation (Figure S1 in Supplementary Materials) by the compound in presence of morphine. These differences could be explained by the observed differences in interaction of compound 1 and compound 2 with the allosteric site in presence of morphine, with compound 2 engaging in more stable interaction, as depicted in Tables S1 and S2. Interestingly, stabilization of particular TM6 conformation seems to play role in modulation of morphine activity by compounds 1 and 2, but not in independent action of compound **1** (Figure 8).

## 4. Materials and Methods

### 4.1. Behavioral Studies

#### 4.1.1. Animals

The experiments were performed on male Albino Swiss mice (18–24 g). 18–10 animals were kept in a cage, at room temperature of 22 ± 1 °C, on a natural dark-light cycle (lights on at 8 a.m.) with free access to food (LSM, Motycz, Poland) and water. All behavioral experiments were carried out according to the National Institute of Health Guidelines for the Care and Use of Laboratory Animals and to the European Community Directive for the Care and Use of Laboratory Animals of 24 November 1986 (86/609/EEC), and approved by the Local Ethics Committee for Animal Experimentation (License No 1009/2012.).

#### 4.1.2. Drug Administration

In behavioral experiments, compounds **1** and **2** obtained, according to previously reported methodology [16,21], were suspended in aqueous solution of 0.5% methylcellulose (tylose) and were injected subcutaneously (s.c.), 60 min before the tests. Morphine (as hydrochloride, Polfa, Kutno, Poland) and cyprodime (CYP, provided by prof. Helmut Schmidhammer from Innsbruck University, Innsbruck, Austria) were diluted in saline and administered subcutaneously (s.c.), 25 min before the test. Acetic acid (0.6% solution) was administered i.p. immediately before observation. All substances were given in a volume of 0.1 mL per 10 g body mass. Animals were weighed immediately before injection. Each experimental group consisted of 10–12 animals per dose and all the animals were used only once. The control animals received an equivalent volume of corresponding vehicle at the respective time before the tests.

#### 4.1.3. Nociceptive Reactions

Nociceptive reactions were studied in the acetic acid ‘writhing’ test [29], and the number of writhing episodes was measured for 10 min, starting 5 min after i.p. administration of acetic acid solution (w = 0.6%) [37]. In this study, the influence of CYP on the antinociceptive effect of the compounds was assessed. Furthermore, morphine at the threshold dose of 0.5 mg/kg, was concomitantly administered with the new derivatives. The dose and time administration of morphine (0.5 mg/kg s.c.) and CYP (2 mg/kg, s.c.) was chosen based on the pilot study (Figures S2 and S3 in Supplementary Materials) and previous results [38,39].

#### 4.1.4. Statistics

The results were calculated by one-way ANOVA followed by Bonferroni’s post hoc test. The results are presented as mean ± standard errors (SEM). A level of *p* < 0.05 was considered to be statistically significant. All figures were prepared using GraphPad Prism version 5.00 for Windows, GraphPad Software (San Diego, CA, USA), www.graphpad.com.

### 4.2. In Silico Studies

Simulations were prepared as described in detail earlier [23]. Summarizing, ligand structures were obtained from The Cambridge Crystallographic Data Centre (CCDC) [40] whenever possible (i.e., morphine, (R)-methadone, naloxone). In absence of a given structure in CCDC (compounds **1** and **2**), it was prepared and optimized with DFT B3LYP 6-31G* basis set in Spartan 10 v. 1.0.1 [41]. Tautomerism and protonation state of the ligands were calculated with LigPrep [42] and Epik [43] programs from Schrödinger suite. In case of compound **1**, the 3-oxo tautomeric form was more stable than 3-hydroxy, which was established experimentally in the previous studies [21], and therefore this form was used in the in silico part of the study. Notably, position of chlorine substituent may affect prevalent tautomeric form in compound **1** derivatives [21]. Protein protonation state was calculated in H++ server [44]. Docking simulations were performed with Surflex implemented in Sybyl 2.0 [45] and with Glide [46]. The active-state μ opioid receptor structure in complex with G protein [47] was immersed in a raft-like membrane (25% 1-palmitoyl-oleoyl-sn-glycero-phosphocholine, POPC; 25% 1-palmitoyl-2-oleoyl-sn-glycero-3-phosphatidylethanolamine, POPE; 30% cholesterol; 20% sphingomyelin) [31], using CHARMM-GUI Membrane Builder [48]. Molecular dynamics simulations were performed in Gromacs [49], using Amber03 force field [50] for protein, Slipids force field [51] for lipids, and General Amber Force Field [52] for ligands. All of these force fields are compatible [53]. TIP3P water model was used, and the NaCl concentration was set to 0.15M. ESP charges for ligands were obtained with R.E.D. Server [54], and ligand topologies were obtained in ACPYPE [55]. Simulations were performed in the NPT ensemble, with timestep of 2 fs. Equilibrated membrane−protein complexes obtained in our previous studies [22,23] were used. Their equilibration procedure included steepest descent minimization and 1 ns NVT simulation, followed by 50 ns NPT simulation, with the protein restrained by a force constant of 10 000 kJ/mol nm^2^. Next, the protein was replaced with protein−ligand complexes or the protein apo form. Colliding water molecules, and a sodium ion migrating to Asp 2.50 was removed. The systems were subsequently minimized by steepest descent and equilibrated by 1 ns NVT and 20 ns NPT simulation steps, with protein and ligands restrained by force constant initially set to 1000 kJ/mol nm^2^, and gradually decreased to 100 kJ/mol nm^2^. All protein-ligand complexes were simulated in triplicate to assure general nature of observations. Principal Component Analysis was performed with gmx covar and gmx anaeig tools of Gromacs. Side chain dihedrals were computed with gmx angle, TM7 rotation was calculated with gmx helixorient. VMD [56] and PyMol [57] were used for visualization of the results. In total, 7.6 μs of all-atom unbiased MD simulation trajectories were analyzed, which is summarized in Table 1.

## 5. Conclusions

In vivo and in silico results obtained in this study are compatible. The investigated compounds are able to enhance antinociceptive activity of morphine, as well as exert such activity on their own. Reversal of activity of compound **1** by CYP suggests direct interactions with MOR, which is in line with previous studies, providing its EC_50_ value to MOR of 14 µM. Different behavior of compound **2** can result from binding to other targets. However, considering possible allosteric mechanism, it can also result from allosteric probe dependence of the compound. The results suggest that compound **1** could serve as a lead in search for novel allosteric agonists, while compound **2** can be developed as a prototypical positive allosteric modulator.

## Figures and Tables

**Table 1:** Summary of trajectories from all-atom, unbiased molecular dynamics simulations analyzed in this work.

Ligands	Abbreviation	Source	Simulation Time	No. of Replicas
No ligands	Apo-MOR	[23]	0.4 μs	1
(R)-methadone	RME	[23]	0.4 μs	3
Morphine	MRF	New simulation	0.4 μs	3
Morphine + compound **1**	MRF+C_1	New simulation	0.4 μs	3
Morphine + compound **2**	MRF+C_2	New simulation	0.4 μs	3
Compound **1**	C_1	New simulation	0.4 μs	3
Naloxone	NLX	New simulation	0.4 μs	3

## Supplementary Materials

Supplementary Materials can be found at https://www.mdpi.com/1422-0067/21/22/8463/s1.

## Abbreviations

BZD	Benzodiazepine
CYP	Cyprodime
DFT	Density functional theory
ECL	Extracellular loop
ESP	Electrostatic Potential
GPCR	G protein-coupled receptor
i.p.	Intraperitoneal
LD	Lethal dose
MD	Molecular dynamics
MOR	μ opioid receptor
PAM	Positive allosteric modulator
PC	Principal Component
PCA	Principal Component Analysis
POPC	1-Palmitoyl-oleoyl-sn-glycero-phosphocholine
POPE	1-Palmitoyl-2-oleoyl-sn-glycero-3-phosphatidylethanolamine
s.c.	Subcutaneously
TM	Transmembrane
TRV-130	Oliceridine
