# Supplementary material for: Novel Positive Allosteric Modulators of µ Opioid Receptor—Insight from In Silico and In Vivo Studies
Source: Int J Mol Sci. 2020 Nov 11;21(22):8463. doi: 10.3390/ijms21228463 (PMC7697543; doi:10.3390/ijms21228463)
Supplement: Supplementary file 1 [file ijms-21-08463-s001.pdf]

International Journal of
Molecular Sciences

Supplementary Materials

Novel Positive Allosteric Modulators of μ Opioid Receptor—Insight from In Silico and In Vivo Studies

Damian Bartuzi [1,*], Ewa Kędzierska [2], Agnieszka A. Kaczor [1,3,*], Helmut Schmidhammer [4] and Dariusz Matosiuk [1]

[1] Department of Synthesis and Chemical Technology of Pharmaceutical Substances with Computer Modeling Laboratory, Faculty of Pharmacy, 4A Chodźki St, PL-20093 Lublin, Poland; dariusz.matosiuk@umlub.pl
[2] Department of Pharmacology and Pharmacodynamics, Faculty of Pharmacy, 4A Chodźki St, PL-20093 Lublin, Poland; ewa.kedzierska@umlub.pl
[3] School of Pharmacy, University of Eastern Finland, Yliopistonranta 1, P.O. Box 1627, FI-70211 Kuopio, Finland
[4] Department of Pharmaceutical Chemistry, Institute of Pharmacy and Center for Molecular Biosciences Innsbruck (CMBI), University of Innsbruck, Innrain 80-82, AT-6020 Innsbruck, Austria; helmut.schmidhammer@uibk.ac.at
* Correspondence: damian.bartuzi@gmail.com (D.B.); agnieszka.kaczor@umlub.pl (A.A.K.); Tel.: +48-81-4487273 (D.B. & A.A.K.)

Figures S1–S3

Tables S1–S4

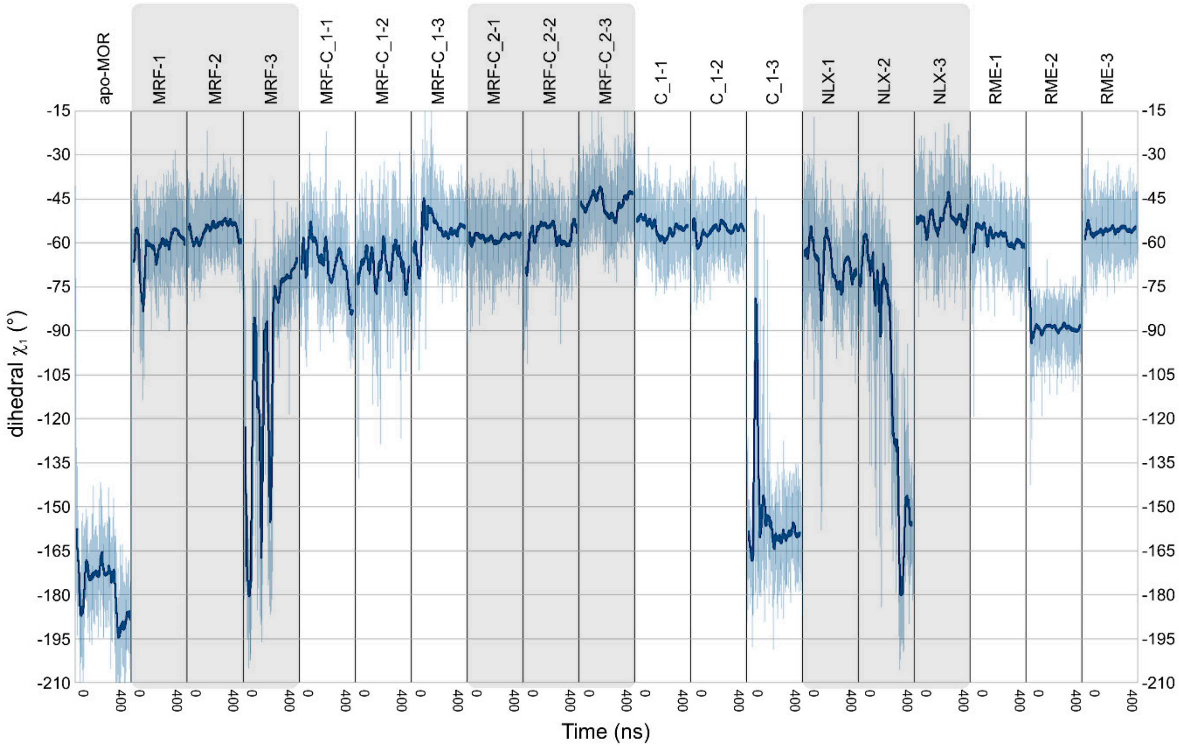

Figure S1. Values of χ_1 dihedral of Tyr 7.53 residue in all trajectories.

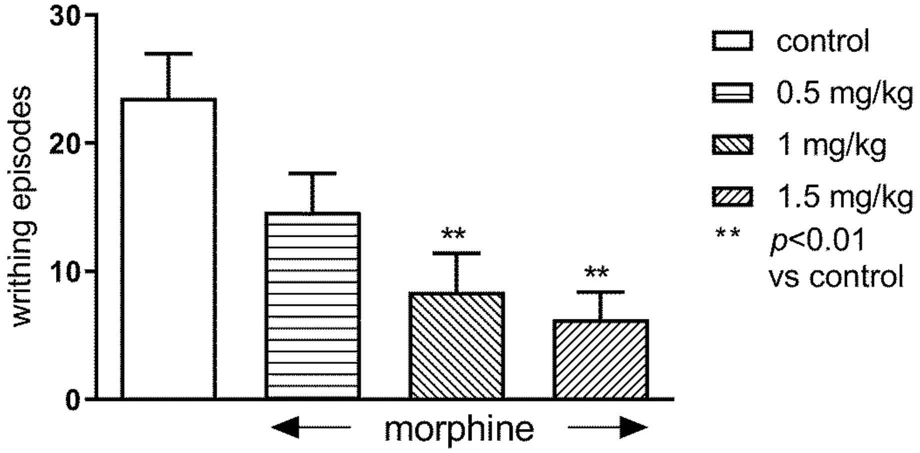

Figure S2. The effect of morphine (05, 1 and 2 mg/kg) on the nociceptive reactions in the 'writhing' test in mice. Morphine was administered s.c. 25 min before the test. The values represent means ± SEM. One-way ANOVA showed significant changes in the writhing episodes after administration of morphine in above doses $F(3,33) = 6.884$; p=0.001). Bonferroni's post hoc test confirmed a significant reduction in writhing episodes after the administration of morphine at the dose of 1 mg/kg (p< 0.001) and 1.5 mg/kg (p<0.01).

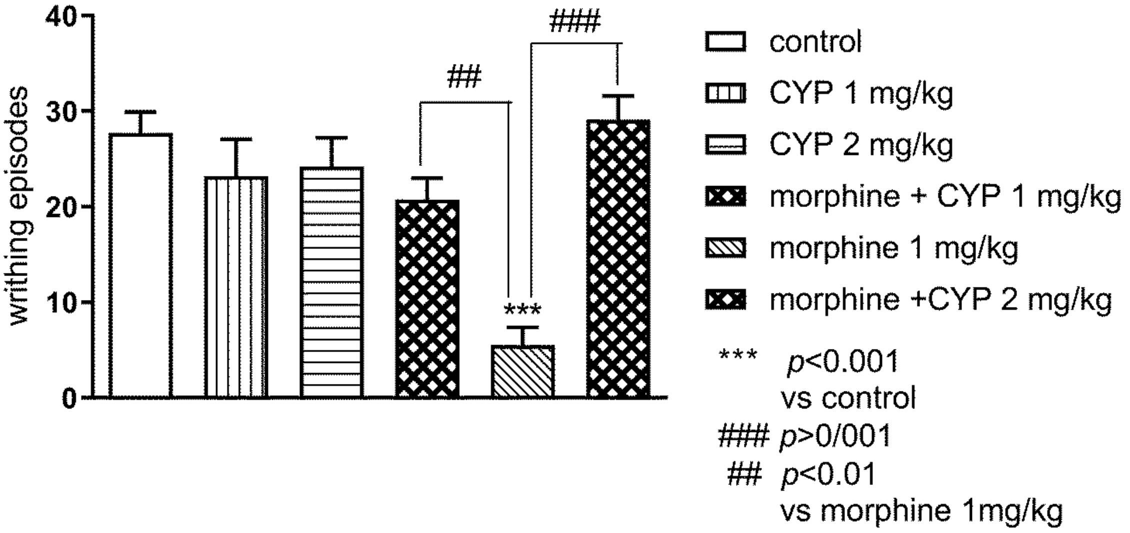

Figure S3. The influence of cyprodime (CYP, 1 and 2 mg/kg) on the antinociceptive activity of morphine in the 'writhing' test in mice. Morphine (1 mg/kg) and CYP were administered s.c. 25 min before the test. The values represent means ± SEM. One-way ANOVA showed significant changes in the writhing episodes after administration of these compounds ($F(5,51) = 10.05$; $p<0.0001$). Bonferroni's post hoc test confirmed a significant reduction in writhing episodes after the administration of morphine at the dose of 1 mg/kg ($p< 0.001$). CYP reversed antinociceptive effect of morphine at the dose 1 mg/kg ($p<0.01$) and at the dose 2 mg/kg ($p<0.001$).

Table S1. RMSF of the investigated compounds calculated in simulations in presence of morphine.

	Compound 1 (nm)	Compound 2 (nm)
Replica 1	0.2301	0.2283
Replica 2	0.2340	0.0961
Replica 3	0.1173	0.0969

Table S2. Ligand RMSD calculated for compound **1** and compound **2** in presence of morphine. Cα atoms of the main chain of the TM7 bundle were used for fitting. Plots show RMSD between a particular frame and a frame 500 ps before. Peaks represent changes of conformation compared to the frame 500 ps before.

	Compound 1	Compound 2
Replica 1	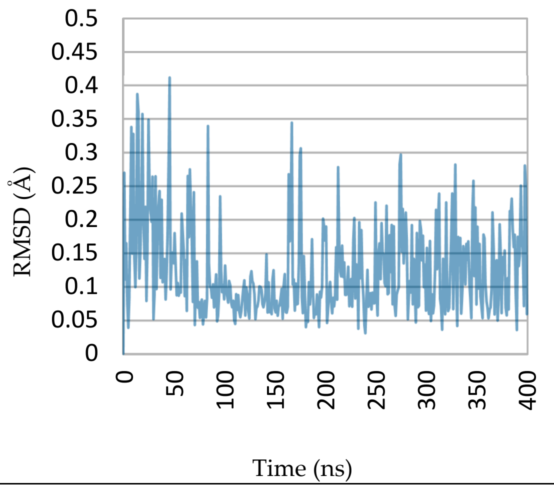	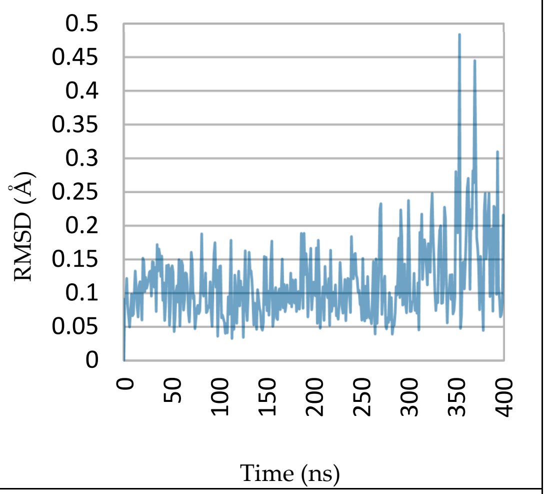
Replica 2	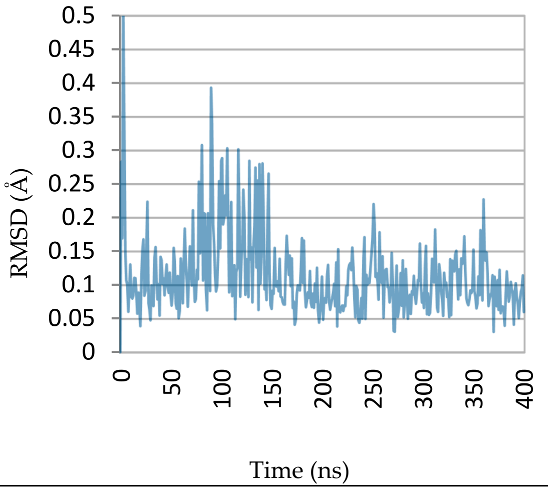	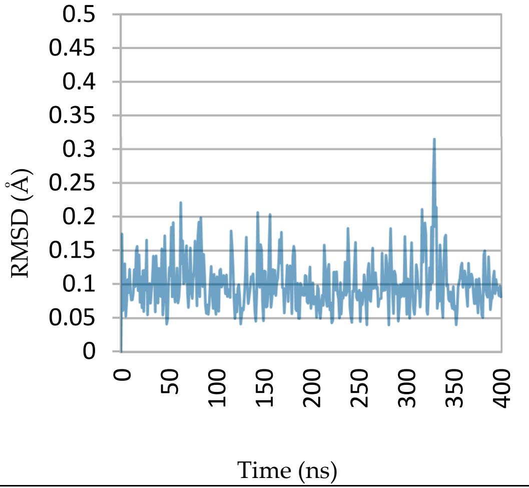
Replica 3	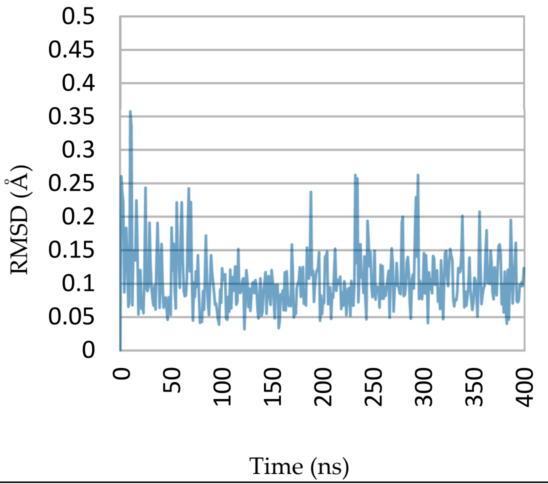	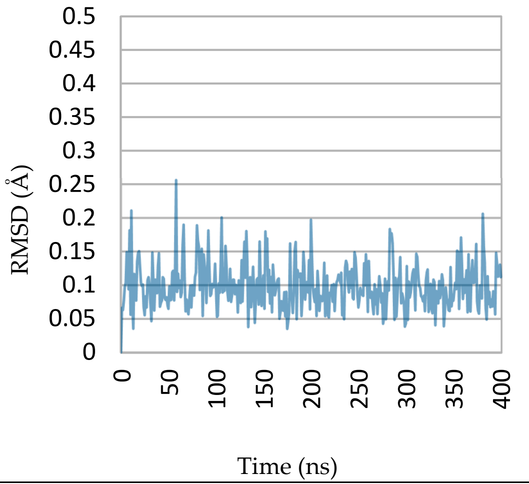

Table S3. Distributions of Tyr 2.64 χ_1 dihedral in all simulations.

	Replica 1	Replica 2	Replica 3
Apo	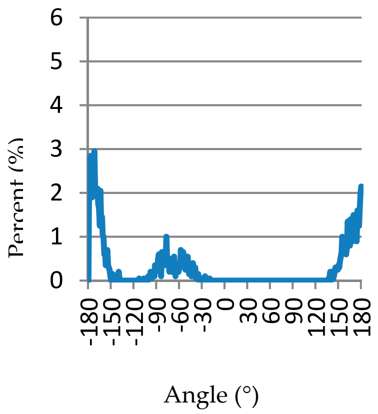		
MRF	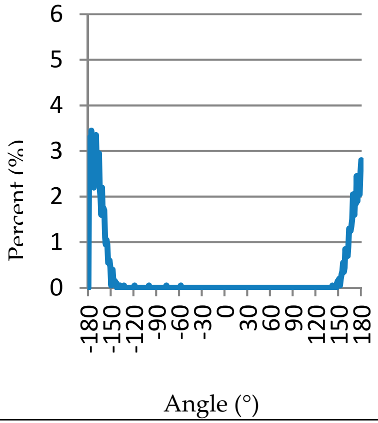	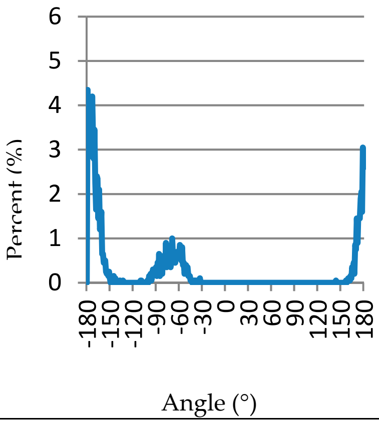	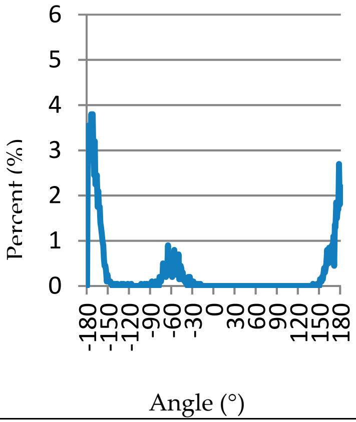
MRF-C_1	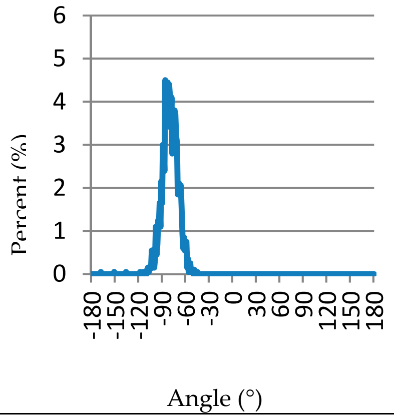	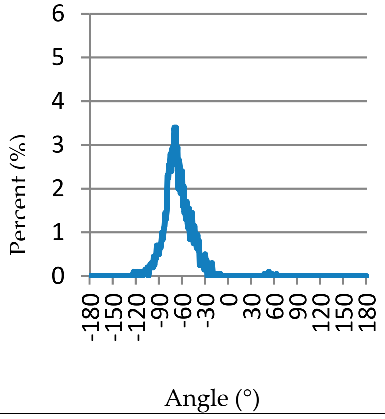	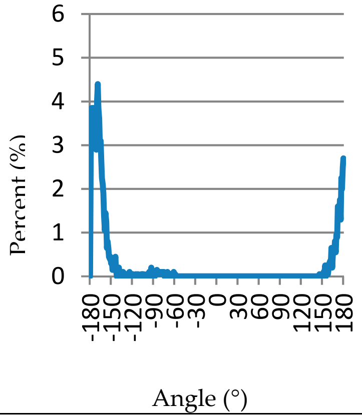

MRF-C 2	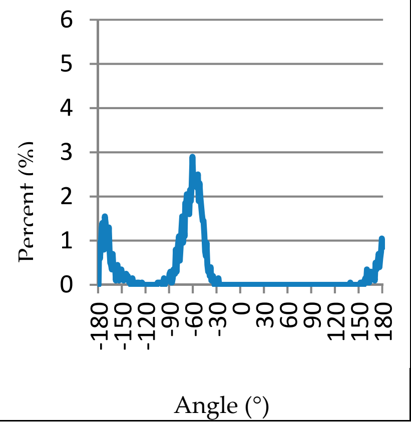	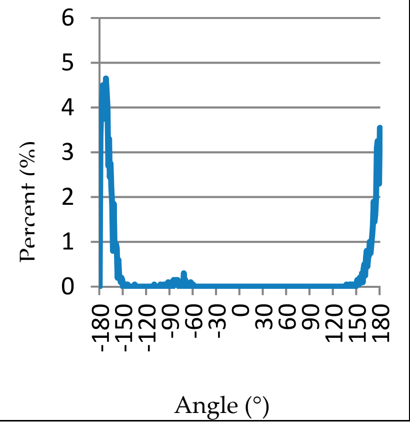	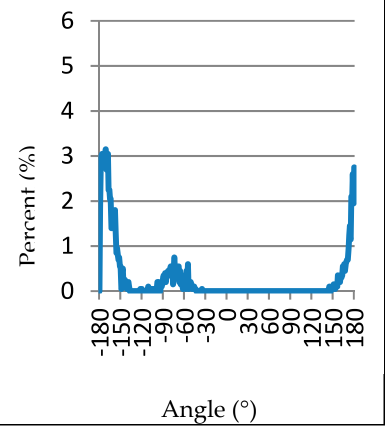

Table S3. *(continued)*

C 1	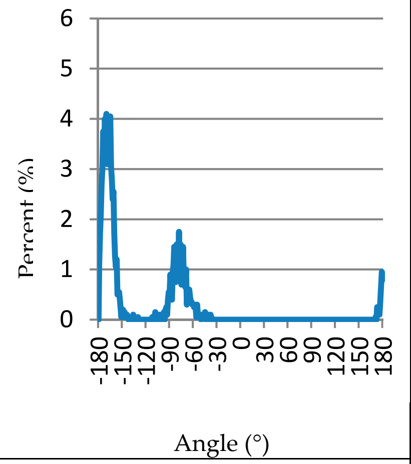	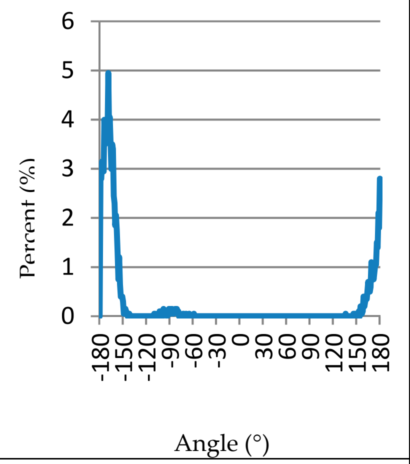	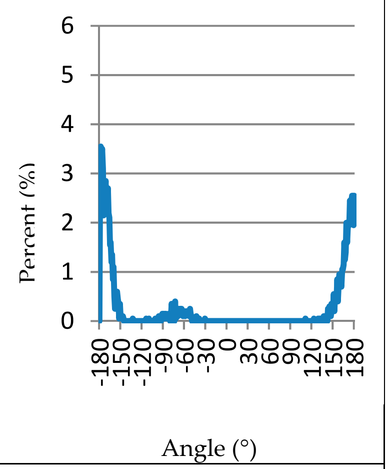
NLX	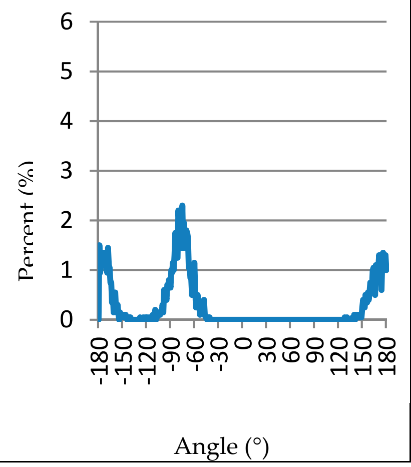	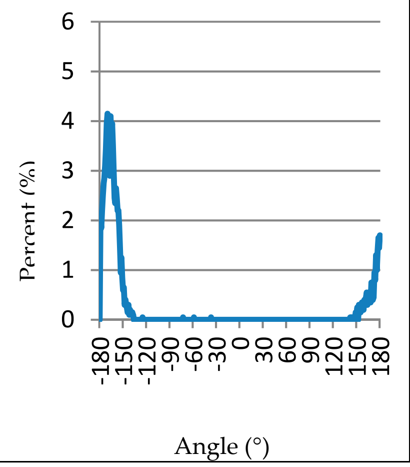	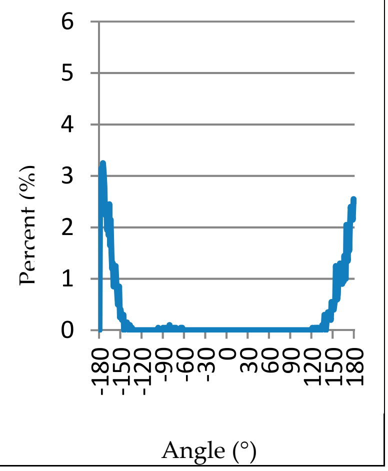

RME	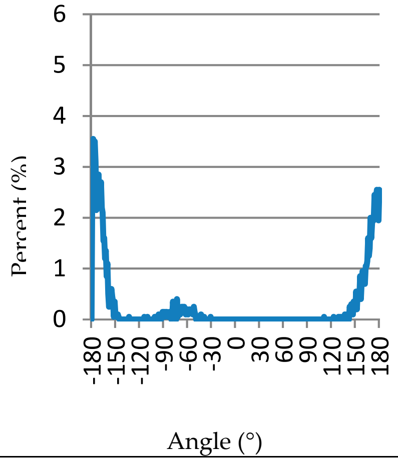	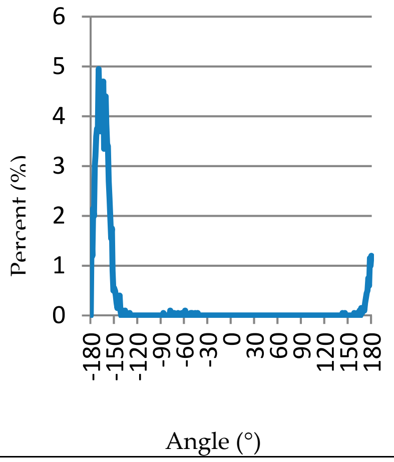	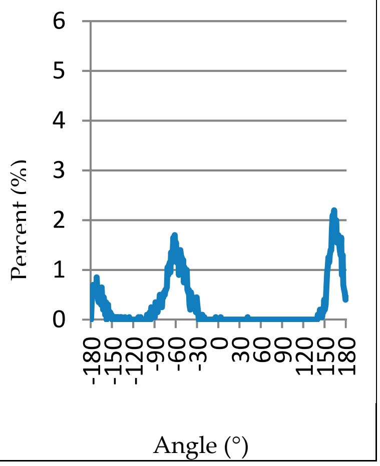

Table S4. Distributions of TM7 angle of rotation in all simulations.

	Replica 1	Replica 2	Replica 3
Apo	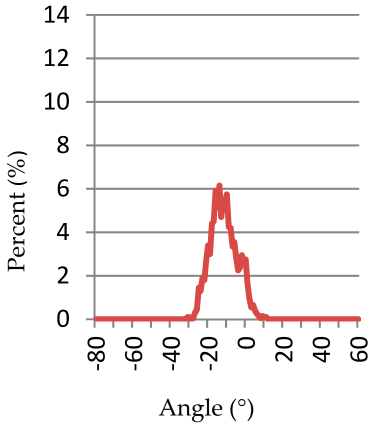		
MRF	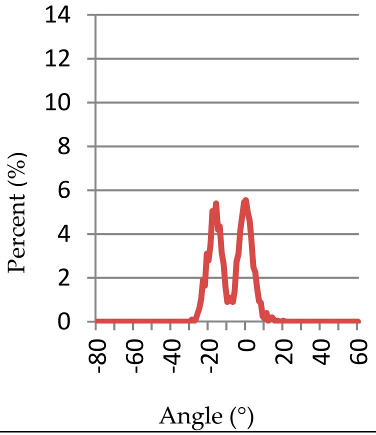	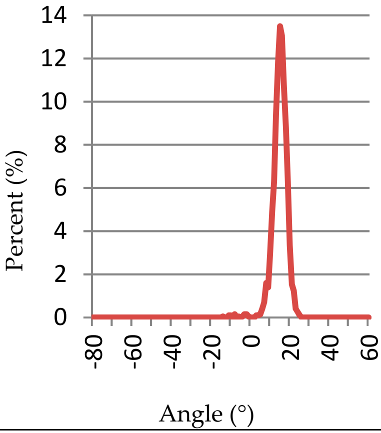	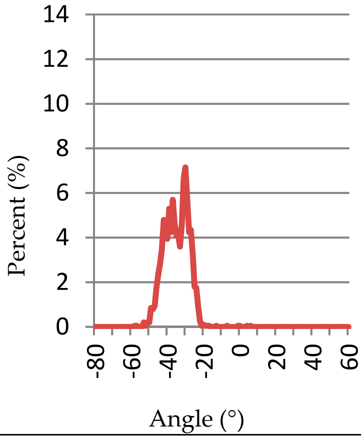

MRF-C_1

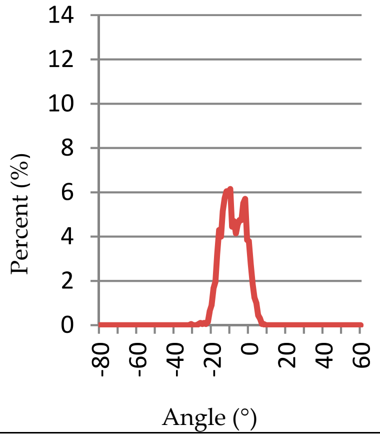

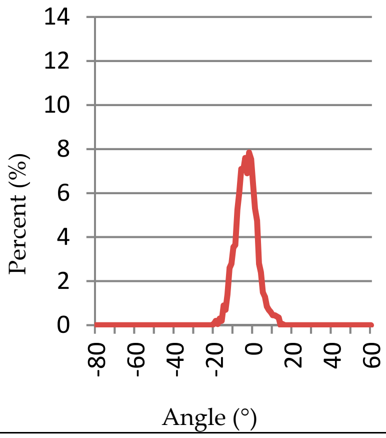

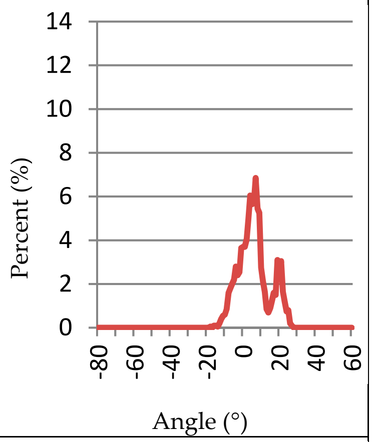

MRF-C_2

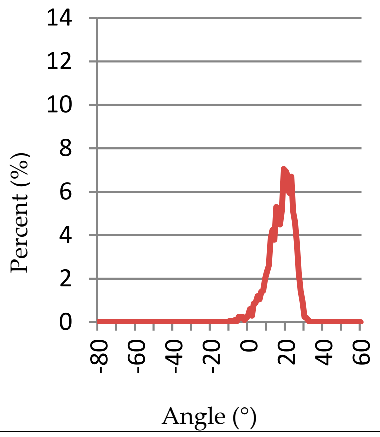

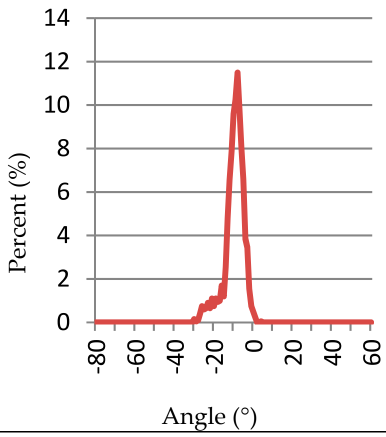

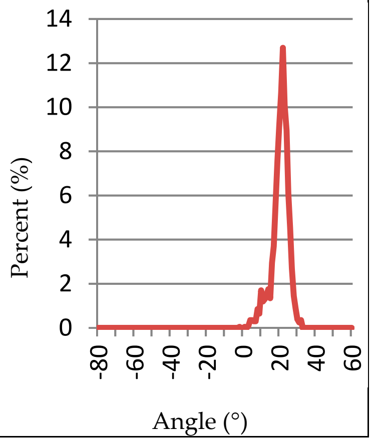

Table S4. *(continued)*

C_1

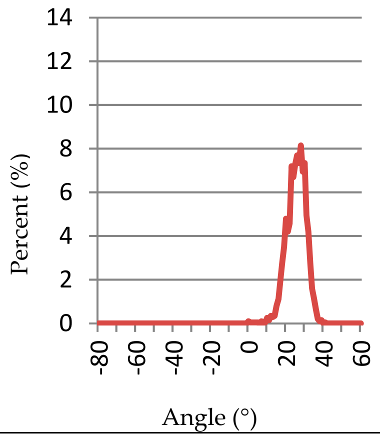

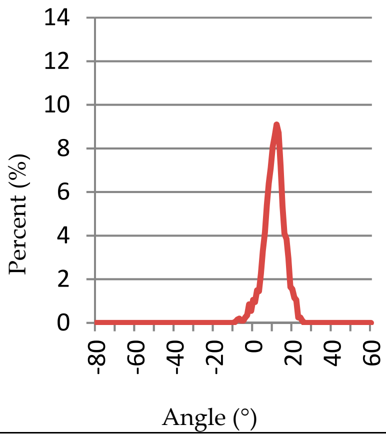

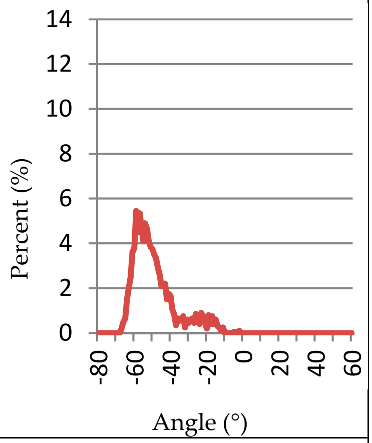

NLX

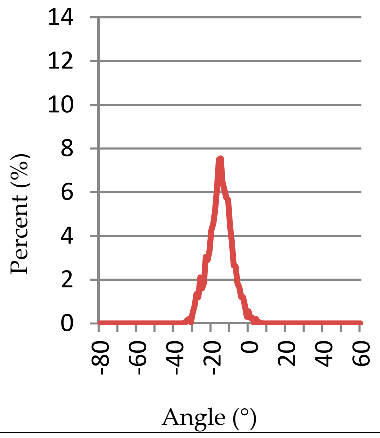

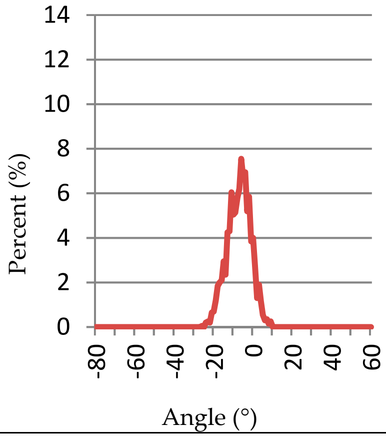

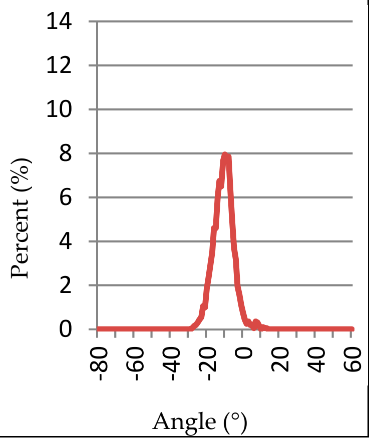

RME

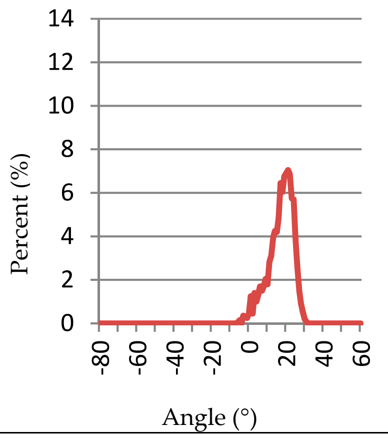
Percent (%)
0 2 4 6 8 10 12 14
-80 -60 -40 -20 0 20 40 60
Angle (°)

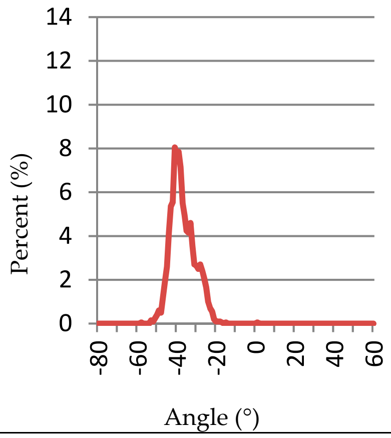
Percent (%)
0 2 4 6 8 10 12 14
-80 -60 -40 -20 0 20 40 60
Angle (°)

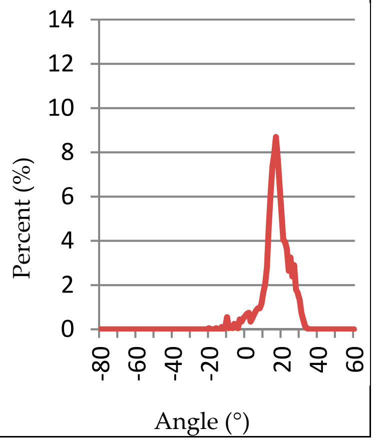
Percent (%)
0 2 4 6 8 10 12 14
-80 -60 -40 -20 0 20 40 60
Angle (°)
